# Highly efficient site-specific transgenesis in cancer cell lines

**DOI:** 10.1186/1476-4598-11-89

**Published:** 2012-12-11

**Authors:** Iacovos P Michael, Claudio Monetti, Anthony C Chiu, Puzheng Zhang, Takeshi Baba, Koichiro Nishino, Siamak Agha-Mohammadi, Knut Woltjen, Hoon-Ki Sung, Andras Nagy

**Affiliations:** 1 Samuel Lunenfeld Research Institute, Mount Sinai Hospital, Toronto, Ontario, M5G 1X5, Canada; 2 Hillman Cancer Center, University of Pittsburgh Medical Center, Pittsburgh, PA, 15261, USA; 3 Department of Obstetrics & Gynaecology, University of Toronto, Toronto, Ontario, M5S 1A8, Canada

**Keywords:** PhiC31 integrase, Site-specific integration, Doxycycline-inducible

## Abstract

**Background:**

Transgenes introduced into cancer cell lines serve as powerful tools for identification of genes involved in cancer. However, the random nature of genomic integration site of a transgene highly influences the fidelity, reliability and level of its expression. In order to alleviate this bottleneck, we characterized the potential utility of a novel PhiC31 integrase-mediated site-specific insertion system (PhiC31-IMSI) for introduction of transgenes into a pre-inserted docking site in the genome of cancer cells.

**Methods:**

According to this system, a “docking-site” was first randomly inserted into human cancer cell lines and clones with a single copy were selected. Subsequently, an “incoming” vector containing the gene of interest was specifically inserted in the docking-site using PhiC31.

**Results:**

Using the Pc-3 and SKOV-3 cancer cell lines, we showed that transgene insertion is reproducible and reliable. Furthermore, the selection system ensured that all surviving stable transgenic lines harbored the correct integration site. We demonstrated that the expression levels of reporter genes, such as green fluorescent protein and luciferase, from the same locus were comparable among sister, isogenic clones. Using *in vivo* xenograft studies, we showed that the genetically altered cancer cell lines retain the properties of the parental line. To achieve temporal control of transgene expression, we coupled our insertion strategy with the doxycycline inducible system and demonstrated tight regulation of the expression of the antiangiogenic molecule sFlt-1-Fc in Pc-3 cells. Furthermore, we introduced the luciferase gene into the insertion cassette allowing for possible live imaging of cancer cells in transplantation assays. We also generated a series of Gateway cloning-compatible intermediate cassettes ready for high-throughput cloning of transgenes and demonstrated that PhiC31-IMSI can be achieved in a high throughput 96-well plate format.

**Conclusions:**

The novel PhiC31-IMSI system described in this study represents a powerful tool that can facilitate the characterization of cancer-related genes.

## Introduction

Mutations and polymorphisms in various genes and/or their regulatory elements are implicated in tumor initiation, progression and drug resistance [1-4]. Elucidation of the consequences of these genetic changes relies largely on the availability of genetic and cancer models. Stable transgene expression is one of the most powerful and informative genetic tools. The generation of stable lines by random integration of a transgene is appropriate when examining the effect of a single transgene. However, when a comparative analysis of a series of transgenes is required, the generation of stable lines using random integration is inefficient. Foremost, expression levels between clones vary significantly due to chromosomal position effects and frequent copy number variation [5]. Thus, the screening and identification of various stable lines with the desired characteristics is extremely laborious. In addition, random integration may lead to genome alterations, such as inactivation of endogenous genes, which may alter cellular phenotype. Inserting the transgene to a predetermined genomic locus, site-specific integration, can eliminate all of the aforementioned confounding factors.

Site-specific integration can be achieved by homologous recombination-based targeting of a specific genomic position [6-9], by recombinase-mediated cassette exchange (RMCE) [10] or integrase-mediated site-specific insertion (IMSI) [11-13]. Homologous recombination, although widely used for gene targeting in mouse embryonic stem cells [14], becomes laborious, time consuming and inefficient in case of human cell lines, due to difficulties involving the design of targeting vectors and the lack of isogenicity required for efficient homologous recombination [9,15,16]. Furthermore, in the case of cancer cell lines, homologous recombination becomes even more difficult due to the fact that these cells acquire many genetic alterations during tumorigenesis [17]. These aberrations further increase during *in vitro* propagation since cancer cells are inherently genetically unstable [18]. Recently, alternative techniques, such as the use of recombinant adeno-associated virus and the introduction of double-strand breaks for stimulation of targeting have been developed, however the targeting efficiency remains very low, generally between 1% and 5%, and often even lower [19-21].

RMCE and IMSI overcome the aforementioned limitations, allowing for both higher efficiency and easier vector construction [11,12]. Current methods involve the introduction of the transgene using bacterial or yeast DNA recombinases into a pre-generated chromosomal locus. The majority of the described strategies use the tyrosine recombinases Cre or Flp and their recognition sites loxP and FRT, respectively [22-26]. Although Cre recombinase has the advantage of being more efficient, it does share a disadvantageous feature with Flp in that both are bi-directional. Since they recognize their recombination products as a substrate, the inserted transgene is often subsequently excised again [27]. In order to overcome this limitation, chimeric Cre recombinases or mutated loxP sites have been generated, such that the recombination products cannot be recognized by Cre recombinase [28-31]. This however, has resulted in decreased efficiency of recombination [29]. An alternative solution is to utilize integrases that only facilitate the insertion reaction.

Phage PhiC31 integrase catalyzes unidirectional recombination between two heterotypic sites, *attP* and *attB* (attachment site for Phage/Bacteria [32]); therefore, this enzyme is the desired candidate to facilitate the insertion of a foreign expression cassette into a precise locus. The resulting sites, *attL* and *attR*, are no longer substrates for PhiC31, thus the integration reaction is not reversible [33,34]. PhiC31 has previously been used for integration of an *attB*-containing plasmid into *attP* sites pre-inserted into the mammalian genome [35-37]. Studies have also shown that pseudo *attP* sites exist in both the human and mouse genomes, and that it is possible to achieve PhiC31-mediated integration into these sites as well [35,36,38].

In this study, we characterized our unique IMSI system that utilizes the PhiC31 integrase (PhiC31-IMSI) for specific integration of the transgene in a pre-inserted genomic landing site (docking site) in various cancer cell lines. We demonstrate that the selection system has 100% fidelity and that sister clones express comparable levels of various reporter genes. In addition, we coupled the PhiC31-IMSI to a doxycycline inducible system allowing for tight and robust control of the expression of the transgene. We also introduced the luciferase gene that could be used for *in vivo* live imaging. Finally, we constructed a series of Gateway compatible intermediate incoming vectors and showed that this system can be applied for high-throughput applications as well.

## Materials and methods

### Plasmid vector construction

**Figure 1:**
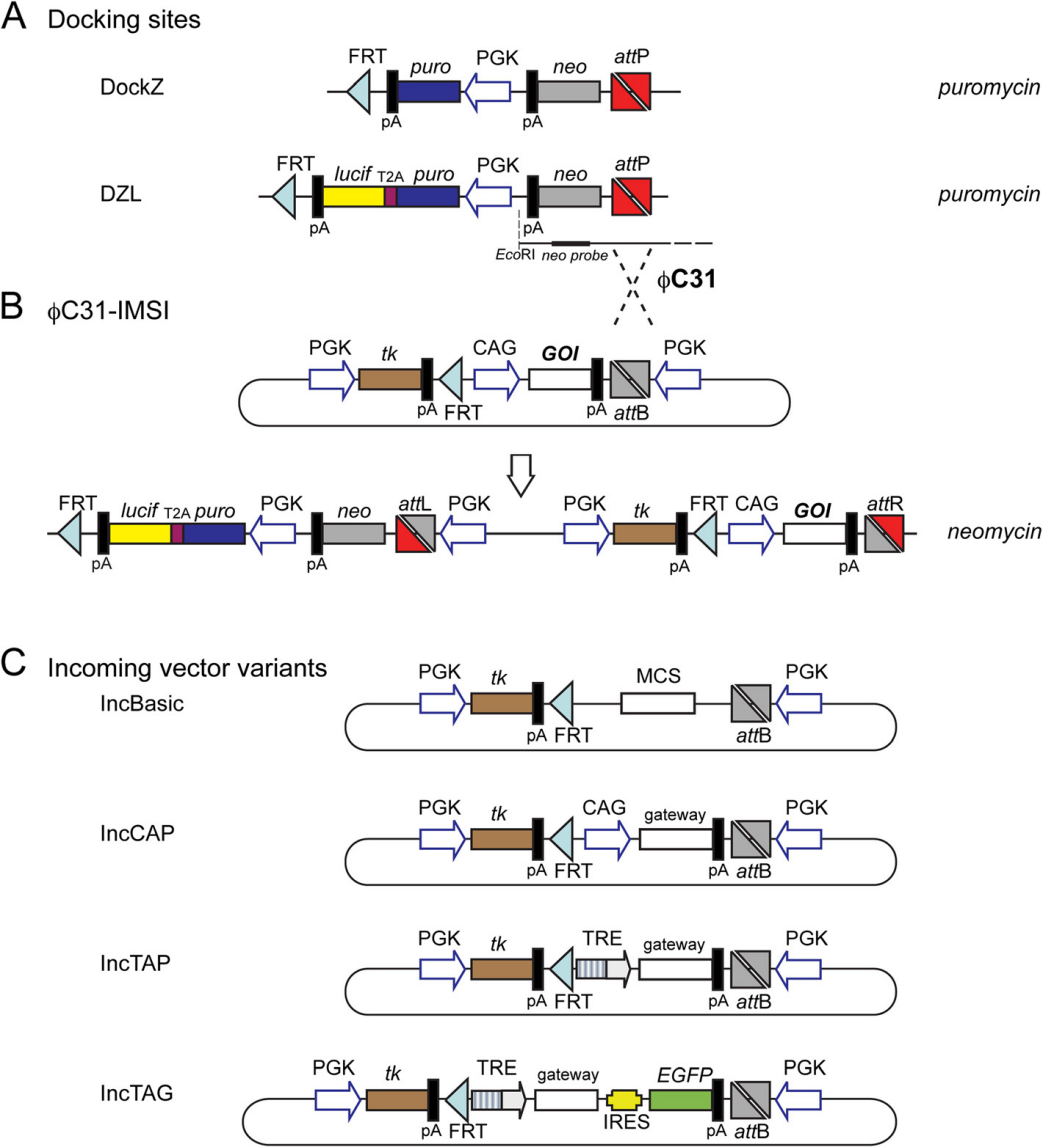
**Docking-incoming system.** (**A**) The basic docking site (DockZ) was modified to contain luciferase as a fusion transgene with puromycin N-acetyl-transferase (PAC), deriving DZL. (**B**) PhiC31 integrase-mediated site-specific insertion of the incoming vector. Correct integrants are selected based on resistance to neomycin. (**C**) A series of incoming vectors with different promoters and reporter genes. IncBasic is promoter-less and contains a multiple cloning site (MCS). IncCAP contains the pCAGGs promoter allowing for constitutive expression of the transgene as well as the Gateway cassette with reading frame A (RfA). IncTAP and IncTAG contain the second-generation tetracycline-regulated promoter (TRE) allowing for inducible expression. Inc-TAG allows for indirect monitoring of the expression of the transgene through a bicistronic arrangement with an IRES followed by EGFP.

We modified our basic docking site vector (DockZ) was described by Monetti *et al.*[39]. This new vector designated as DZL contains a fusion gene comprising puromycin N-acetyl-transferase (PAC) and luciferase linked via the T2A peptide [40], *i.e.* PAC-T2A-luciferase (Figure 1A). The T2A junction along with part of the C-terminus of PAC and N-terminus of luciferase was synthesized and cloned into pBluescript between *Bst*API and *Cla*I sites. This fragment was then subcloned into DockZ using *Bst*API and *Cla*I. The C-terminus of luciferase was subcloned from a pBluescript (pBS) luciferase-containing vector after digestion with *Bsr*GI and *Eco*NI.

The basic incoming vector, Inc-basic, as well as Inc-CAG-MCS-pA and Inc-CAG-EGFP-pA were described by Monetti *et al. *[39] and shown in Figure 1B. After digestion of Inc-CAG-MCS-pA with *Hpa*I, the Gateway RfA cassette was inserted to generate Inc-CAG-Gateway-pA. Inc-TRE-EGFP-pA was constructed by excision of the pCAGGs promoter and insertion of second-generation tetracycline-regulated promoters, TRE [41] by *Bam*HI and *Hind*III digestion. Subsequent excision of EGFP by *Bsu*36I and *Nhe*I and cloning of MCS gave rise to Inc-TRE-MCS-pA. The Gateway RfA cassette was inserted after *Hpa*I digestion in order to generate Inc-TRE-Gateway-pA. The IRES-EGFP digested with *Eco*RI (polished) and *Cla*I was inserted into Inc-TRE-MCS-pA after *Bsu*36I (polished) and *Cla*I digestion generating Inc-TRE-MCS-IRES-EGFP-pA. The Gateway RfA cassette was inserted after *Hpa*I digestion, constructing Inc-TRE-Gateway-IRES-EGFP-pA.

The reverse-transactivator of tetracycline [rtTA; Tet-ON Advance [42] was subcloned from the pTet-On Advanced vector (Clontech) into pBluescript using *Eco*RI and *Bam*HI sites. Subsequently, it was subcloned into pcDNA6 digested with *Hind*III and *Sac*II deriving pcDNA6-rtTA.

### Cell culture

The cancer cell lines Pc-3 (prostate cancer), Du145 (prostate cancer) and SKOV-3 (ovarian cancer) were purchased from ATCC and maintained in RPMI-1640 media containing 10% fetal bovine serum (Invitrogen). Cells were transfected with plasmids using ExGen500 (Fermentas) according to the manufacturer's protocol. Stable clones were selected using 1.0 μg/ml puromycin (Sigma), 7.0 μg/mL blasticidin (Sigma) and 750 μg/mL G418 (Invitrogen). 2.0 μg/mL of doxycycline was used to induce gene expression under the control of the TRE promoter. Cell lines were incubated at 37°C with 5% CO_2_.

### Generation and screening of stable cell lines

Stables lines for DockZ and DZL were derived by transfection of 10^6^ cells (Pc-3 for DockZ; DU145 and SKOV-3 for DZL) with 10 μg DNA linearized with *Eam*1105I. Forty-eight hours after transfection, cells were trypsinized and replated at limited dilutions. Resistant colonies were selected with puromycin and picked using cloning cylinders after two weeks of selection.

Single-copy stable lines were screened by southern blot. Ten micrograms of genomic DNA was digested with *Eco*RI overnight, resolved by gel electrophoresis and transferred to Hybond N+ (GE Healthcare). Single copy integrants were detected using a neomycin phosphotransferase (neo^R^) probe.

### PhiC31-mediated recombination and selection for IMSI derivatives

**Figure 2:**
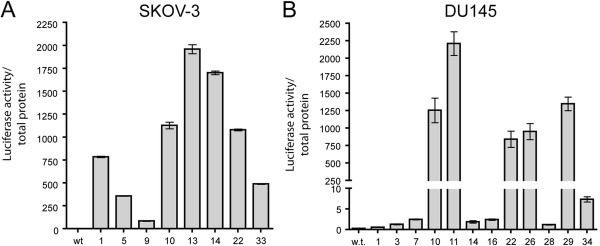
**Characterization of luciferase containing docking site (DZL).** Normalized luciferase levels of single-copy DZL-containing clones of SKOV-3 (**A**, n=3) and DU145 lines (**B**, n=3).

Recombination was performed by cotransfection of 10^6^ cells with 7.5 μg of incoming vector and 2.5 μg of pCAGGS-PhiC31 using ExGen 500 (Fermentas). Forty-eight hours after transfection, resistant colonies were selected with G418. For 96-well format PhiC31-mediated integration, 0.66 μg of incoming vector and 0.33 μg of pCAGGS-PhiC31 were used. Correct integrants were verified with standard PCR using primers recognizing the *attL* site (CCAGGGCGTGCCCTTGAGTTCTCTC) and neo^R^ gene (CGATGAATCCAGAAAAGCGGCCATTTTTC) and/or southern using the thymidine kinase probe (Figure 2A).

### Reporter assays

The Luciferase assay (Promega) was performed according to the manufacturer’s protocol. In brief, cells grown on 24-well plates were washed twice with phosphate-buffered saline (PBS) and incubated in 200 μl lysis buffer for 30 min on ice. Twenty microliters of cell lysate was mixed with 25 μl of luciferase reagent and luciferase activity was read on the luminometer. Luciferase activity was normalized to total protein measured using the BCA kit (Pierce).

The FACSAria™ cell sorter (BD biosciences) was used for single cell analysis of fluorescent expression. Cells grown on 6-well dishes were trypsinized and suspended in PBS. 7-AAD or propidium iodide, diluted 1:100 in PBS, was used for detection of apoptotic cells in the case of GFP and dsRed stably transfected cells, respectively. Ten thousands cells were analyzed per sample.

Soluble Flt1-Fc and free human VEGF were measured using commercially available sandwich ELISAs (R&D systems, catalog # MVR100 and DVE00, respectively) following the manufacturer’s protocol.

### Xenograft assays

For xenograft assays, 5×10^6^ cells were suspended into 150 μl RPMI-1640 media containing 33% of matrigel and injected subcutaneously in both dorsal flanks of SCID mice. Doxycycline was administered using doxycycline-containing pellets (0.625g/kg, Harlan Laboratories). Tumour size was monitored using calipers and the volume was calculated using the formula V= (LxWxH)π/6.

## Results

### PhiC31-mediated site-specific transgene integration system: validation of its fidelity in cancer cell lines

In this study, we modified our recently developed PhiC31 integrase-mediated transgene insertion docking site, DockZ [39] to derive DZL (Figure 1A). Both docking sites allow for introduction of a transgene in their predetermined genomic integration of the desired cell line. Cell with docking site insertion can be identified with puromycin selection. The docking site also carries an inverted promoter-less neomycin phosphotransferase (neo^R^) gene at the 5’ end of the *attP* site. The transgene is inserted with the incoming vector through PhiC31-mediated integration. The incoming vector contains an *attB* site (Figure 1B) that is subjected to recombination along with the *attP* site of the docking site. Selection for the correct integrants is achieved through activation of the promoter-less neo^R^ gene in the docking site. The incoming vector contains a promoter that becomes positioned upstream of the neo^R^ gene upon correct integration and starts its expression (Figure 1B). In addition, the original system allows for removal of all selection markers through Flp recombinase-mediated deletion [39] and negative selection for removal of the thymidine kinase gene by means of selection for FIAU resistance (Figure 1B). In our cancer cell lines, however, we did not perform this resolution step since our aim was to take advantage of a built-in positive and negative selectable marker system for future drug selection for or against the cancer cells. In the DZL docking site we added a luciferase transgene linked to the puromycin N-acetyl-transferase, in a bicistronic manner through a T2A peptide [40]. Thus the luciferase allows *in vivo* imaging of tumours. Both proteins were expected to remain functional despite the addition of the T2A peptide resulted 10 amino acids at the carboxy terminus of PAC and a proline at the amino terminus of luciferase genes. To test the docking site and establish the properties of this system, we used a series of incoming vectors (Figure 1C) and three human cancer cell lines (Pc-3, DU145 and SKOV-3).

After establishing stable lines for the docking site transgene, single transgene insertion colonies were identified by Southern blotting using a neo^R^ probe on genomic DNA cut with the diagnostic *Eco*R1 restriction enzyme (Figure 1A). For the Pc-3 line, a total of 36 clones were screened, three of which contained a single copy of DockZ (data not shown). For the SKOV-3 and DU145 lines, 8 out of 48 and 12 out of 48 clones, respectively, contained a single copy of DZL (data not shown), showing that the PAC was active. All single-copy containing clones showed the same proliferation rate as the original lines, indicating that the insertion did not have any detrimental effects (data not shown). Furthermore, we performed the luciferase assay, demonstrating that the luciferase gene was also active in those clones (Figure 2A and 2B).

**Figure 3:**
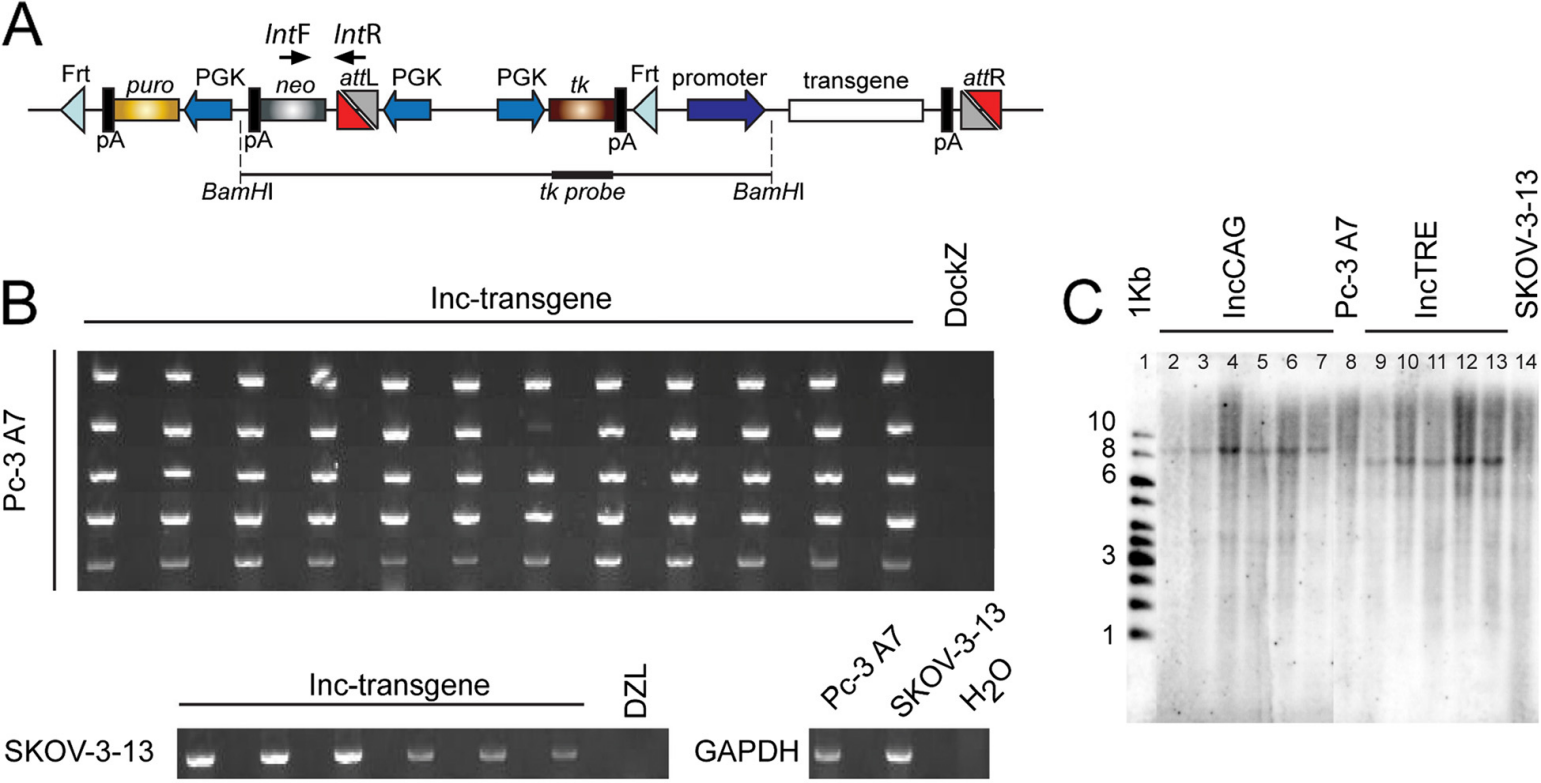
**Fidelity of the docking-incoming system.** (**A**) Position of the primer pairs used for screening of integration (*Int*F and *Int*R) as well as the *tk* probe used for southern analysis. (**B**) PCR amplification of the integration junction using primers recognizing the *att*L site and the neo^R^ probe. Sixty-six colonies (60 colonies for Pc-3-A7 and 6 for SKOV-3-13) were screened and all had the correct integration site. Analysis was done using a multicomp agarose gel. (**C**) Southern blot analysis of subclones derived by integration of two different incoming vectors (IncCAG-transgene; lanes 2–7, and IncTRE-transgene; lanes 9–13) into line Pc-3-A7 (lanes 2–7) and SKOV-3-13 (lanes 9–13). Genomic DNA was digested with *BamH*I and the *tk* probe was used. Original Pc-3-A7 and SKOV3-13 DockZ lines were also included in lanes 8 and 14, respectively. 1-Kb marker is shown in the first lane.

Single-copy docking site clones were screened for PhiC31-mediated integration of an incoming vector encoding for EGFP. Integration was successful for one out of three clones for the Pc-3 line (*i.e.* Pc-3-A7) and two out of eight for the SKOV-3 line (*i.e.* SKOV-3-13 and SKOV-3-33). We were not able to achieve successful integration in any of the DU145 DZL lines. Subsequently, these clones were subjected to PhiC31-mediated integration of various incoming vectors and the fidelity of the integration was characterized. A total of 66 colonies (60 for Pc-3-A7 and 6 for SKOV-3-13) were analyzed for correct integration using pairs of PCR primers specific to the *attL* site and the neo^R^ gene (Figure 3A). The expected 700 bp fragment was successfully amplified in all screened colonies (Figure 3B), proving that all integration events into the docking sites occurred in a correct manner. Furthermore, 6 Pc-3-A7 and 5 SKOV-3-13 colonies for IncCAG and IncTRE, respectively, were also analyzed by Southern blot using a *tk* probe in order to demonstrate single integration of the incoming vector (Figure 3A). A single band corresponding to the expected size (9.2Kb and 7.5Kb for Inc-CAG and Inc-TRE, respectively) was detected; indicating that after PhiC31-mediated integration, a single recombination event between the a*ttP* site of the docking site and the a*ttB* site of the incoming vector occurred (Figure 3C).

### Uniform expression of the transgenes

**Figure 4:**
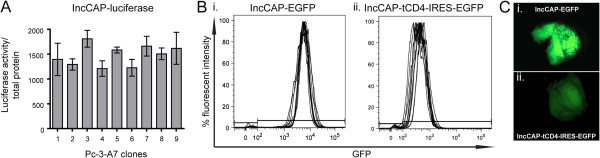
**Expression of reporter genes from isogenic clones.** (**A**) Normalized luciferase levels of nine isogenic clones derived after integration of an incoming luciferase containing plasmid under the control of pCAGGs (IncCAP-luciferase) into line Pc-3-A7. There was no significant difference in luciferase activity in isogenic clones (*P*=0.509, n=3). Error bars show the standard deviation of the mean. (**B**) Histogram plots of EGFP expression of ten isogenic clones derived after integration of (i) IncCAP-EGFP and (ii) IncCAP- tCD4-IRES-EGFP into the Pc-3-A7. The GFP levels were an order of magnitude higher (Table 1) when EGFP was expressed as a single gene. (**C**) Fluorescent images of xenografts derived from Pc-3-A7 lines stably integrated with (i) IncCAP-EGFP and (ii) IncCAP-tCD4-IRES-EGFP. The relative ratio of EGFP levels of the two vectors was maintained *in vivo* as well.

We used two different reporter assays in order to examine the expression levels between clones derived from the same parental line. The incoming vector, expressing luciferase under the control of pCAGGs, was introduced into the DockZ docking site containing Pc-3-A7 line and nine clones were isolated. Luciferase activity was measured and normalized to total protein (Figure 4A). One-way ANOVA analysis indicated that the expression levels were similar between isogenic clones (*P*=0.509) in contrast to the remarkable expression differences observed between SKOV-3 and DU145 lines (Figure 2A and 2B) with different genomic integration for the luciferase transgene.

In order to further examine the expression between isogenic clones, we used an incoming vector encoding EGFP or dsRed under the control of pCAGGs either as a single or bicistronic gene, coupled with an IRES to a truncated form of CD4 (tCD4), missing the intracellular and extracellular domains responsible for interaction with other proteins (I. P. Michael and A. Nagy, unpublished data) (Figure 4B). Using flow cytometry for single cell analysis, we assessed the GFP expression after a period of 2-month continuous culture of isogenic clones. We showed uniform GFP expression in each clone (Figure 4Bi and 4Bii, Additional file 1: Figure S1) as well as a similar mean fluorescent value between isogenic clones derived from Pc-3-A7 and SKOV-3-13 (Table 1 and data not shown). Furthermore, essentially all cells (99%) were positive for GFP (Table 1).

**Table 1:** Characterization of EGFP expression of isogenic clones

**GFP**			**IRES-EGFP**		
**Clone**	**GFP+ median**	**% GFP +**	**Clone**	**GFP+ median**	**% GFP +**
1	5970	99.2	1	461	98.7
2	5492	99.4	2	440	99.5
3	5655	98.5	3	502	99.3
4	5512	99.2	4	446	98.8
5	6751	99.6	5	538	99.5
6	5332	99.7	6	386	99.2
7	5990	99.5	7	359	98.7
8	5290	99.2	8	358	98.3
9	7031	99.6	9	371	98.9
10	5122	99.6	10	459	98.9
Average	5814.5	99.35	Average	432.0	98.98

Mean GFP expression was one order of magnitude lower when EGFP was expressed as a bicistronic gene using IRES (Table 1). After establishing xenografts, we were able to show that the difference in expression level between pCAGGs EGFP and pCAGGs tCD4-IRES-EGFP was maintained at the tumor site as well (Figures 4Ci and 4Cii). Similar results were obtained when dsRed was used instead of EGFP (data not shown).

### Generation of the doxycycline-inducible transgene expression system

**Figure 5:**
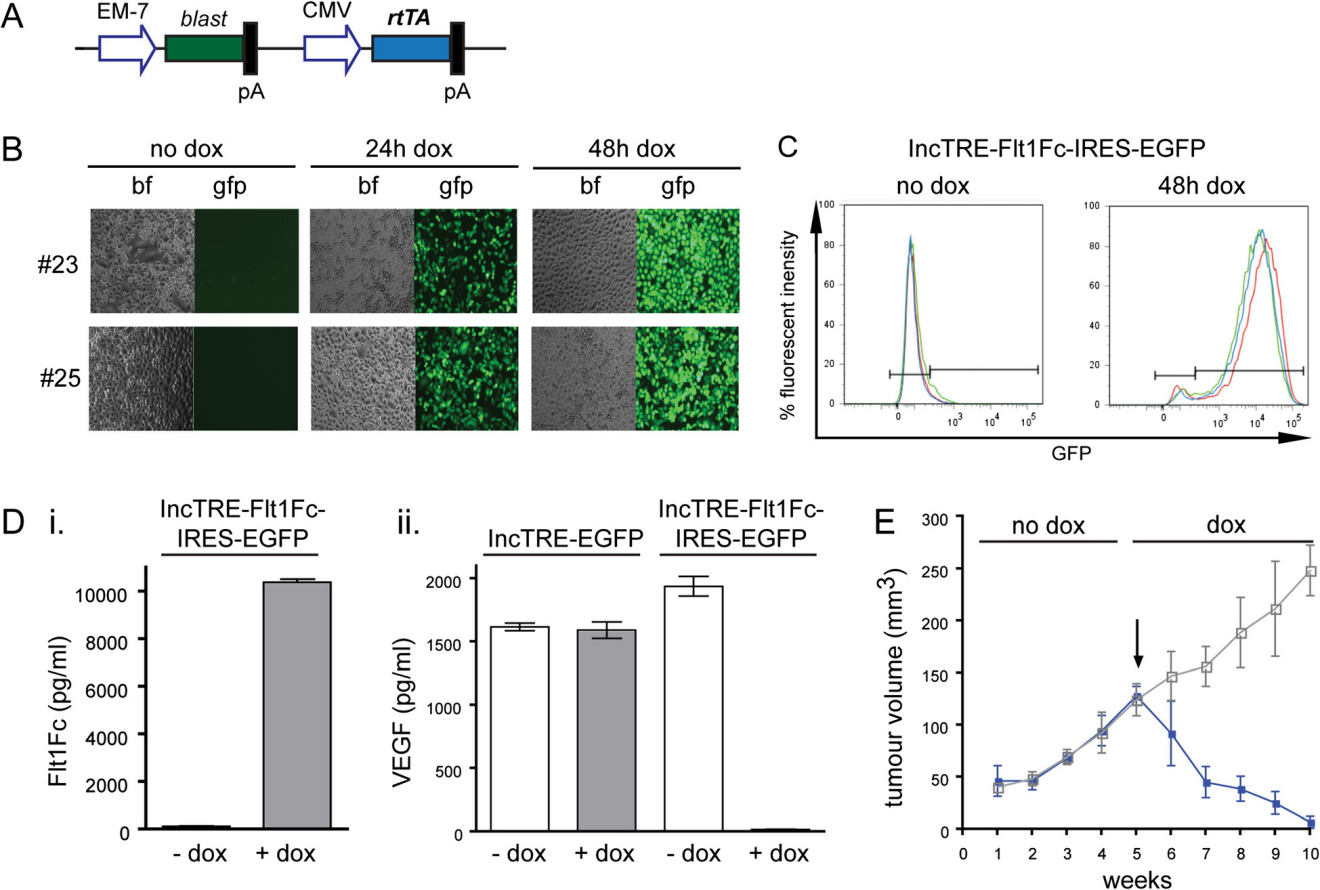
**Generation of an inducible system.** (**A**) Plasmid encoding the second generation of rtTA under the control of CMV. (**B**) EGFP expression after integration of IncTRE-EGFP into Pc-3-A7 rtTA sublines #23 and #25, before and after induction with dox for 24 h and 48 h. bf; brightfield, gfp; fluorescent. (**C**) Histogram plots of EGFP expression of three isogenic clones derived after integration of IncTRE-Flt1-Fc-IRES-EGFP into Pc-3-A7-23. (**D**) Expression levels of (i) Flt1-Fc and (ii) VEGF in the supernatant of Pc-3-A7-23 stably transfected with IncTRE-EGFP or IncTRE-Flt1Fc-IRES-EGFP, before (white columns) and 48h after (grey columns) dox induction (n=3). (**E**) Tumour volume of Pc-3-A7-23 xenografts stably transfected with IncTRE-EGFP (empty grey squares, n=4) or IncTRE-Flt1Fc-IRES-EGFP (filled blue squares, n=4). Arrow indicates the time point at which the mice were switched from normal chow to doxycycline containing food pellets.

To obtain temporal control on transgene expression, we coupled the IMSI strategy with the doxycycline-inducible system. We created a range of incoming vectors containing second-generation tetracycline responsive elements coupled with the minimal CMV promoter (TRE [41]) (Figure 1C). The second-generation reverse tetracycline transactivator was randomly integrated in lines Pc-3-A7 and SKOV-3-13 using linearized pcDNA6-rtTA-Adv (Figure 5A) and clones resistant to blasticidin were selected. An incoming vector for EGFP under the control of TRE was introduced through PhiC31-IMSI. After addition of doxycycline, strong GFP expression was observed for both Pc-3-A7 and SKOV-3-13 rtTA lines, while no GFP was expressed in the absence of doxycycline (Figure 5B and Additional file 1: Figure S2). Withdrawal of doxycycline resulted in the disappearance of GFP expression, indicating that this system is tightly regulated (Additional file 1: Figure S3).

Then an incoming vector, encoding a secreted protein able to trap Vascular Endothelial Growth Factor (VEGF trap), *i.e.* Flt1-Fc [43,44], under the control of the TRE promoter and coupled to EGFP through IRES was stably introduced into line Pc-3-A7-23. Using flow cytometry, we showed that after 72 h of doxycycline administration, uniform expression of GFP was observed in three different sister clones (Figure 5C). Furthermore, using a sensitive ELISA for Flt1-Fc, we demonstrated that its expression was tightly regulated; no protein was detected in the absence of doxycycline while high levels were measured after 72h of dox administration (Figure 5Di). Flt1-Fc can also trap VEGF secreted by Pc-3 cells. By using an ELISA that detects only free VEGF, we were able to show that after 72h of doxycycline treatment and secretion of Flt1-Fc, VEGF was ablated from the supernatant, while no reduction of its levels was observed in the absence of doxycycline (Figure 5Dii). Inducible expression of EGFP alone did not affect the levels of VEGF in the media (Figure 5Dii).

In order to examine if inducibility is maintained *in vivo*, we established xenografts of Pc-3-A7-23 expressing Flt1-Fc or EGFP under the control of TRE. Since previous studies indicate that Flt1-Fc can slow tumour growth through inhibition of angiogenesis [43], we used this as a functional assay to test the stability and inducibility of our system *in vivo*. After establishing xenografts for Pc3-A7-23 transfected with Inc-TRE-EGFP (control) and Pc-3-A7-23 transfected with Inc-TRE-Flt1Fc-IRES-EGFP, no difference in tumour growth rate was observed during the first five weeks, indicating that tight regulation is maintained (Figure 5E). After inducing the expression of the transgene by feeding the xenograft bearing animals with doxycycline containing pellets, a rapid reduction in tumour size was observed for Pc-3-A7-23 stably transfected with Flt1-Fc, indicating that inducibility is maintained in the xenograft as well (Figure 5E).

### Feasibility of a high-throughput system

The generation of a series of incoming vectors for derivation of isogenic clones would require both the cloning of GOIs in the desired vector as well as the establishment of stable cell lines. To facilitate and accelerate this procedure, we coupled our system with the Gateway cloning system [45]. We inserted reading frame A (RfA) of the Gateway system into three different incoming vectors, generating Inc-pCAGGs-RfA-pA (Inc-CAP), Inc-TRE-RfA-pA (Inc-TAP) and Inc-TRE-RfA-IRES-EGFP-pA (Inc-TAG) (Figure 1C). This allows for high-throughput insertion of cDNA libraries into the desired incoming vector.

We then examined if it was feasible to achieve PhiC31-mediated integration in a 96-well format. Using the Pc-3-A7 clone, we were able to establish stable clones using three different incoming vectors expressing EGFP, dsRed and luciferase. On average, four G418 resistant colonies were observed per well (Table 2 and Additional file 1: Figure S3). All of the colonies transfected with EGFP or dsRed were positive, while the luciferase colonies were not characterized (data not shown). One-way ANOVA analysis indicated that the number of resistant colonies was independent from the transgene that was introduced (*P*=0.298). Out of 48 wells, 47 had at least one resistant colony (98%, Table 2). Similar results were observed when an incoming vector for Flt-1Fc-IRES-EGFP under the control of TRE was introduced in line Pc-3-A7-23, with all colonies expressing EGFP upon addition of doxycycline (data not shown).

**Table 2:** Integration frequency in a 96-well plate format

**Number of colonies per well**			
**Well #**	**EGFP**	**dsRed**	**Luciferase**
1	5	5	3
2	1	3	6
3	3	3	4
4	3	4	3
5	3	6	6
6	0	4	6
7	5	5	6
8	1	3	4
9	4	4	5
10	4	3	6
11	2	3	3
12	3	7	4
13	5	4	4
14	5	4	8
15	7	4	4
16	8	5	2
Average	3.9	4.2	4.6
Positive wells*	93.75%	100%	100%

On average, each well was containing 4.2 resistant colonies. In total, 98% of the wells were containing at least one resistant colony.

## Discussion

In this study, we characterized a PhiC31-IMSI system in cancer cell lines that allows for the control and precise insertion of a transgene in a predetermined genomic locus. To broaden the utility of this system, we combined it with a doxycycline-inducible expression system of the transgene as well as a luciferase reporter that features *in vivo* live imaging in animal models.

The phage serine recombinase PhiC31 is adopted in this system in order to facilitate the integration of a transgene into the host genome. First, a docking site carrying an *attP* site upstream of a promoter-less neo^R^ gene is randomly inserted as a single copy into the host genome by means of selection for puromycin resistance. The incoming vector, carrying an *attB* site in front of a promoter, is subsequently inserted via PhiC31-mediated integration into the genome (Figure 1A). The incoming vector also carries the transgene under the control of the desired promoter. In addition, using the Flp recombinase and thymidine kinase for negative selection, the neo and puro selection markers can be excised leaving behind only a clean integration of the transgene [39]. However, this aspect was not examined in the present study since maintenance of the selection markers offers the advantage for easy isolation of pure populations of cancer cells from tumour models that, for example, resist or develop resistance to various therapeutic schemes. In order to further advance the IMSI, we introduced the expression of luciferase that allows for *in vivo* live imaging and monitoring of tumour growth and metastasis. We implemented this feature by constitutively expressing luciferase as a fusion transgene with the PAC gene of the docking site (Figures 1 and 2). As far as we know, this is the first luciferase-containing IMSI.

Using this system, we were able to show that all of the resistant colonies in two different cancer cell lines, Pc-3 and SKOV-3, were a result of the integration of the incoming vector in the desired genomic locus. The DU145 cancer line was not permissive to PhiC31-mediated integration. Our unpublished data (K. Nishino, A. Nagy) indicate that this might be either the result of methylation of the *Att*P site or due to the fact that the *Att*P is missing as a result of endogenous nuclease activity. Taken our combined experiences with IMSI in cancer and Embryonic Stem cell lines [13,39] we do not expect considerable limitations in the use of this method. In agreement with previous studies describing pseudo-*attP* sites in human cell lines, we did not observe any multiple integrations in the same clone [35,38]. It is possible that integration at pseudo-*attP* sites may occur yet G418 selection selects against these events.

Insertion of various transgenes in the same genomic locus resulted in similar expression levels when isogenic clones were compared (Figure 4). Such reproducible expression allows for various applications, such as the characterization of methylation and transgene orientation on transgene expression, as well as the characterization of promoter and enhancer elements [46-49]. In this study, we compared the levels of EGFP expressed either directly under the control of a strong promoter or as a bicistronic gene under the control of the same promoter. The GFP levels were different in the two cases, with the levels dropping an order of magnitude when EGFP was expressed in a bicistronic fashion under the control of an IRES sequence (Figure 4 and Table 1). We were also able to show that all the aspects of expression levels characterized *in vitro* were maintained after establishment of *in vivo* xenografts, implying that this system is robust and can be utilized for *in vivo* studies (Figure 4).

Many studies involving transgenes require temporal control of their expression. Thus, we coupled our system with the doxycycline inducibility. In addition to the temporal expression, this system also allows for control of the expression levels of the transgene [42]. After creating stable lines of the docking site, we derived sublines constitutively expressing rtTA-Advance [42]. In combination with incoming vectors containing the TRE promoter [41], we showed that this system allows for tight and inducible regulation of EGFP. Furthermore, in a proof-of-principle experiment, we derived stable lines expressing soluble VEGF trap, Flt1-Fc, in a doxycycline inducible manner [43,44]. We showed that the expression of Flt1-Fc was tightly regulated *in vitro.* Furthermore, this tight regulation was also maintained in xenograft assays, where the transgene (in our case Flt1-Fc) could exert its biological activity (Figure 5).

Finally, in an attempt to make this system more flexible, we combined it with Gateway technology (Figure 1 and Table 2). All incoming vectors were made Gateway compatible, which allows for fast and reliable generation of incoming-expression libraries. This, in combination with the fact that this system can be used in a 96-well set-up, could allow for high-throughput generation of isogenic stable lines for the expression of gene libraries.

## Conclusions

Precise control of chromosomal insertion and expression of various genes involved in tumor progression are essential for the creation of future cancer models and to further our understanding of the intriguing maze of biological processes underlying complex diseases as cancer. The IMSI described here offers reliable and directional integration of a transgene into a specific locus of the genome of cancer cell lines utilizing the PhiC31 recombinase. It also allows for reproducible and inducible expression of the transgene as well as derivation of expression libraries in a high-throughput manner. These characteristics were maintained *in vivo* during xenograft assays, which along with its live imaging feature due to luciferase expression, makes this system a powerful tool for broad aspects of cancer research.

## Competing interests

The authors declare that they have no competing interests.

## Authors’ contribution

IPM design the overall study, conducted the majority of experiments, collected and analyzed the data. CM, KN, TB, KW, HS contributed to experimental design. CM, ACC, PZ, HS conducted experiments. SAM offered reagents. AN guided the study. IPM and AN wrote the manuscript. All authors discussed the results and commented on the manuscript. All authors read and approved the final manuscript.

## Supplementary Material

Additional file 1 Highly efficient site-specific transgenesis in cancer cell lines. Click here for file
