# Supplementary material for: Highly efficient site-specific transgenesis in cancer cell lines
Source: Mol Cancer. 2012 Dec 11;11:89. doi: 10.1186/1476-4598-11-89 (PMC3537676; doi:10.1186/1476-4598-11-89)
Supplement: Additional file 1 — Highly efficient site-specific transgenesis in cancer cell lines. [file 1476-4598-11-89-S1.pdf]

Supplemental Information

Highly efficient site-specific transgenesis in cancer cell lines

Supplementary Figure 1: Fluorescent analysis of EGFP expression of three subclones derived after integration of Inc-CAP-EGFP into the SKOV-3-13.

Supplementary Figure 2: Fluorescent analysis of EGFP expression of three subclones derived after integration of Inc-TRE-EGFP into SKOV-3-13 stably transfected with rtTA before and after dox.

Supplementary Figure 3: Dynamics of doxycycline inducibility characterized by EGFP. PC-3-A7-23-EGFP cell culture was exposed to doxycycline for 48hrs. After splitting into two plates, doxycycline was withdrawn from one of them while kept in the other. 96hrs after doxycycline withdrawal, GFP levels were significantly decrease and were almost undetectable 144hrs after withdrawal.

Supplementary Figure 4: Number of resistant colonies after transfection with ΦC31-IMSI incoming vector with EGFP, dsRed and luciferase. There was no statistically significant difference in the number of colonies per well for the three different transgenes (One-way ANOVA, P=0.298).

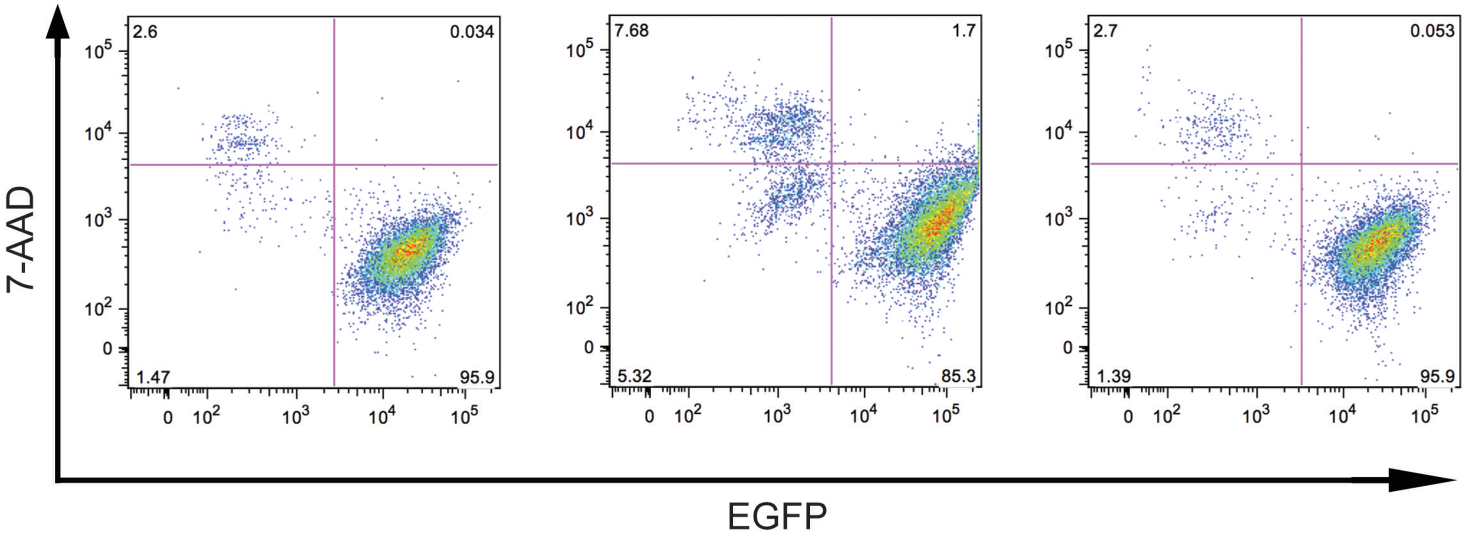

Supplementary Figure 1

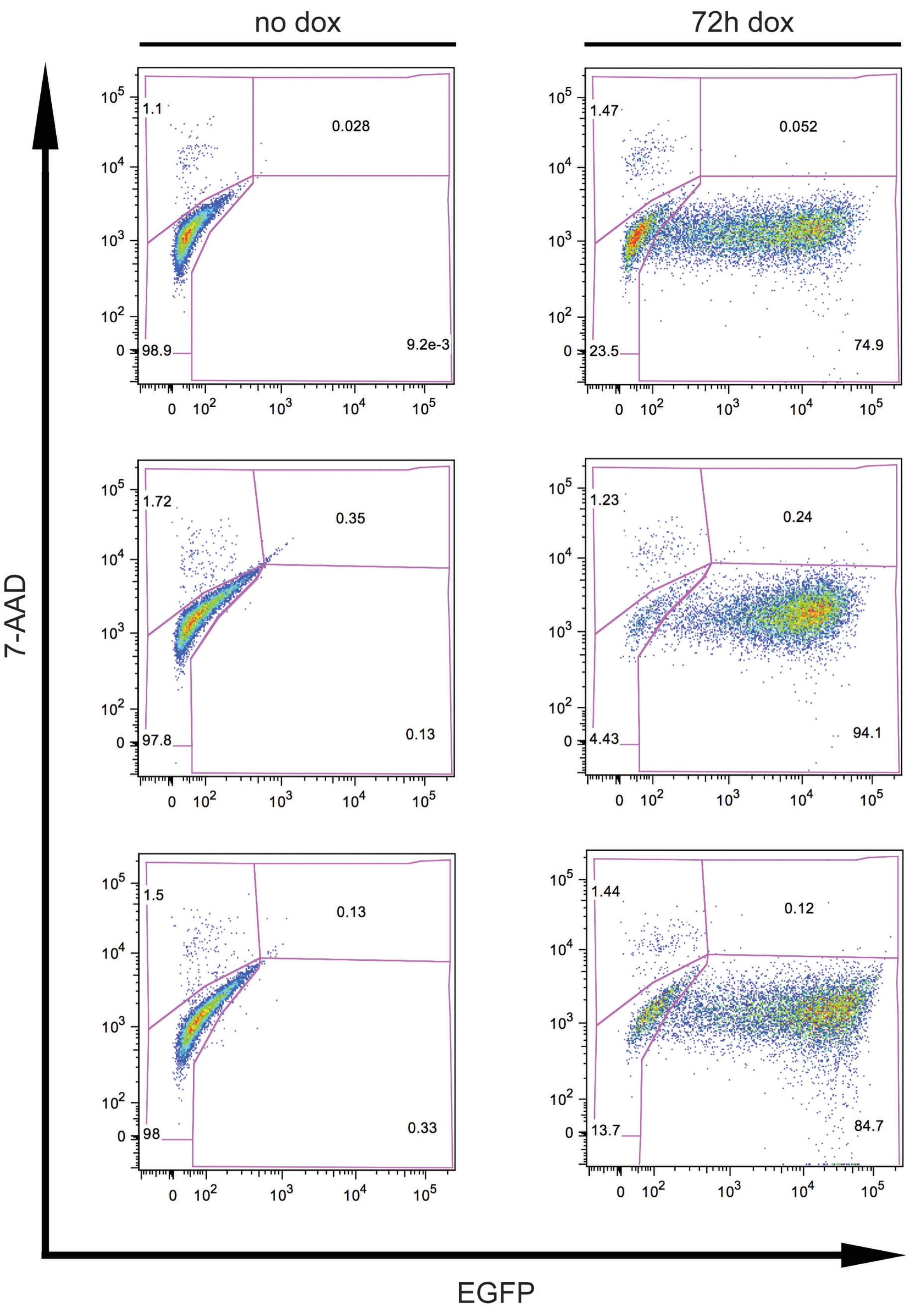

Supplementary Figure 2

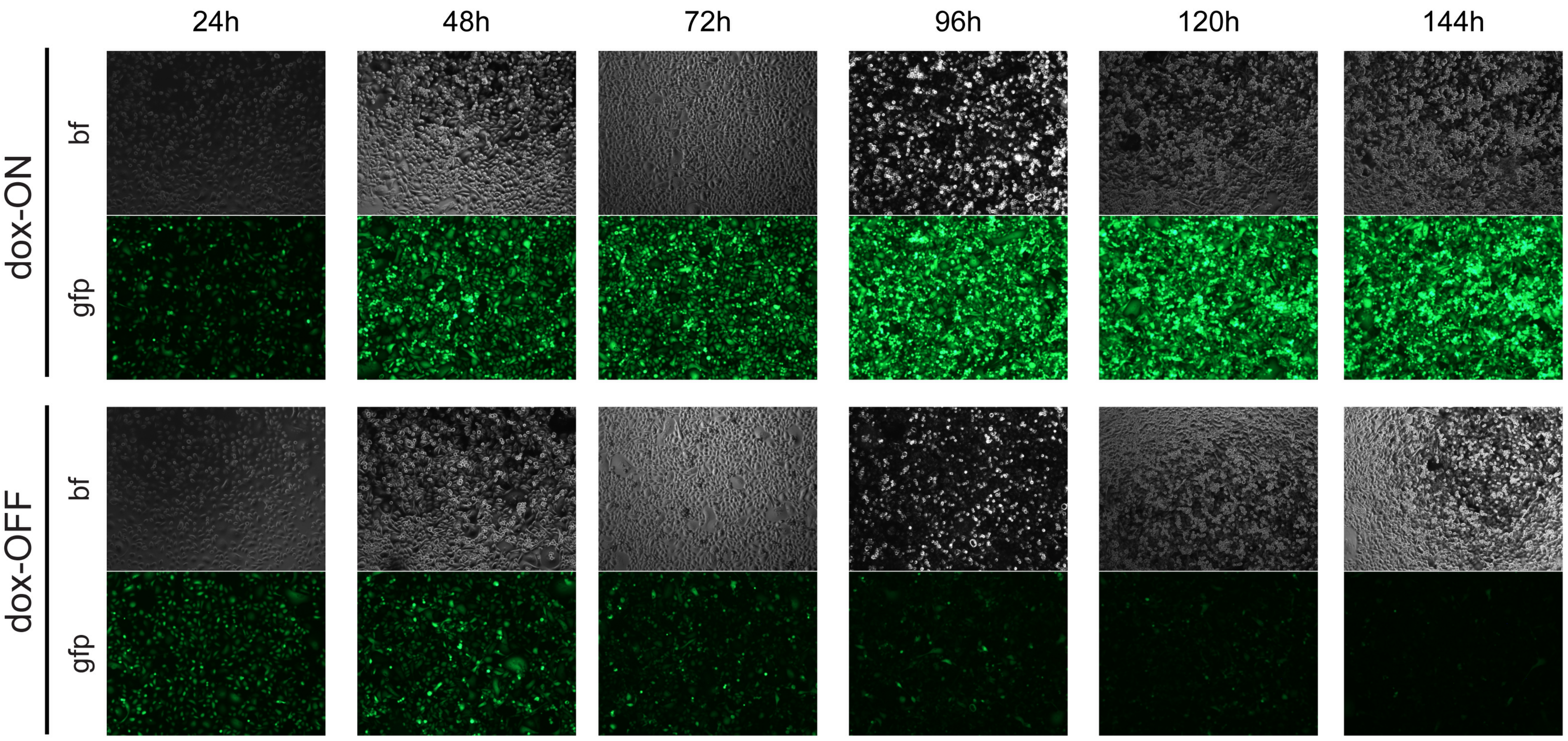

Supplementary Figure 3

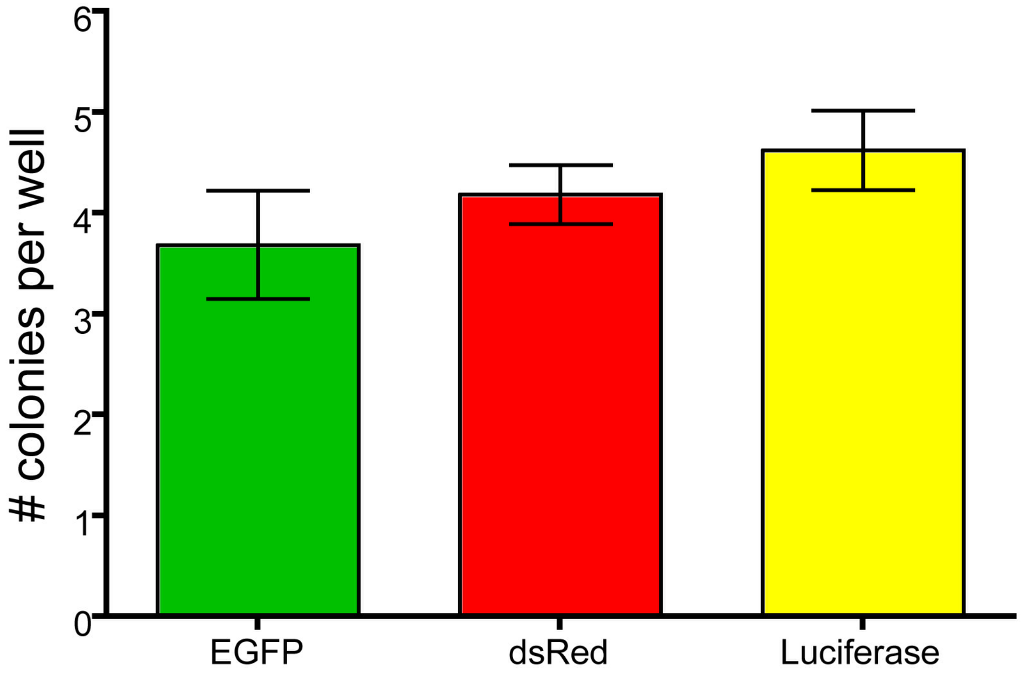

Supplementary Figure 4
